# Preserved Learning during the Symbol–Digit Substitution Test in Patients with Schizophrenia, Age-Matched Controls, and Elderly

**DOI:** 10.3389/fpsyt.2014.00189

**Published:** 2015-01-06

**Authors:** Claudia Cornelis, Livia J. De Picker, Wouter Hulstijn, Glenn Dumont, Maarten Timmers, Luc Janssens, Bernard G. C. Sabbe, Manuel Morrens

**Affiliations:** ^1^Collaborative Antwerp Psychiatric Research Institute, University of Antwerp, Antwerp, Belgium; ^2^University Psychiatric Center St. Norbertushuis, Duffel, Belgium; ^3^Donders Institute for Brain, Cognition and Behaviour, Radboud University Nijmegen, Nijmegen, Netherlands; ^4^Janssen Research and Development, Janssen Pharmaceutica N.V., Beerse, Belgium; ^5^Psychiatric Hospital Broeders Alexianen, Boechout, Belgium

**Keywords:** symbol–digit substitution test, coding task, processing speed, implicit learning, schizophrenia

## Abstract

**Objective:** Speed of processing, one of the main cognitive deficits in schizophrenia is most frequently measured with a digit–symbol-coding test. Performance on this test is additionally affected by writing speed and the rate at which symbol–digit relationships are learned, two factors that may be impaired in schizophrenia. This study aims to investigate the effects of sensorimotor speed, short-term learning, and long-term learning on task performance in schizophrenia. In addition, the study aims to explore differences in learning effects between patients with schizophrenia and elderly individuals.

**Methods:** Patients with schizophrenia (*N* = 30) were compared with age-matched healthy controls (*N* = 30) and healthy elderly volunteers (*N* = 30) during the Symbol–Digit Substitution Test (SDST). The task was administered on a digitizing tablet, allowing precise measurements of the time taken to write each digit (writing time) and the time to decode symbols into their corresponding digits (matching time). The SDST was administered on three separate days (day 1, day 2, day 7). Symbol–digit repetitions during the task represented short-term learning and repeating the task on different days represented long-term learning.

**Results:** The repetition of the same symbol–digit combinations within one test and the repetition of the test over days resulted in significant decreases in matching time. Interestingly, these short-term and long-term learning effects were about equal among the three groups. Individual participants showed a large variation in the rate of short-term learning. In general, patients with schizophrenia had the longest matching time whereas the elderly had the longest writing time. Writing time remained the same over repeated testing.

**Conclusion:** The rate of learning and sensorimotor speed was found to have a substantial influence on the SDST score. However, a large individual variation in learning rate should be taken into account in the interpretation of task scores for processing speed. Equal learning rates among the three groups suggest that unintentional learning in schizophrenia and in the elderly is preserved. These findings are important for the design of rehabilitation programs for schizophrenia.

## Introduction

Schizophrenia is a psychiatric disorder, characterized by positive symptoms (e.g., hallucinations and delusions), negative symptoms (e.g., avolition and reduced emotional expressivity), and severe cognitive disabilities. Since cognitive deficits in schizophrenia are significantly correlated to poor functional outcomes (1) and quality of life (2), the development of pharmacological and remediation techniques addressing these impairments could be highly beneficial to the clinical outcome.

Cognition is not a single entity but can be divided into several domains. In schizophrenia research, the areas of primary interest are: processing speed, attention/vigilance, working memory, verbal learning, visual learning, executive functioning and social cognition (3). The combination of these domains may contribute differently to the overall clinical picture of cognitive decline in schizophrenia. Experimental tasks that focus on isolating the relative influence of these specific cognitive domains are needed to specify which deficits are most pronounced in order to provide a targeted treatment.

Processing speed has been shown to be a very distinguishing and reliable factor to characterize cognitive deficits in schizophrenia (4). This parameter reflects the speed with which different cognitive and sensorimotor functions are executed (5). Viewed from a traditional experimental psychology perspective, processing speed can be conceived as the total sum of three different stages of information processing, namely perceptual analysis, response selection, and response execution (6, 7).

Although there are several neuropsychological tests for measuring reduced processing speed, a recent meta-analysis (8) has demonstrated that a digit–symbol-coding task is the most sensitive test to apply to patients with schizophrenia. Moreover, this meta-analysis identified processing speed impairment as the largest single deficit in the cognitive abilities of schizophrenia (8, 9).

Digit–symbol-coding tasks have been carried out in two different ways. In one version, the Digit–Symbol Substitution Test (DSST), symbols have to be drawn under their corresponding digits according to a key of digit–symbol combinations, provided at the top of the sheet. The second version is the symbol-coding subtest of the Brief Assessment of Cognition in Schizophrenia included in the MATRICS Final Battery (7, 10). This task does not require the drawing of symbols but rather the numerals (1–9) have to be written as quickly as possible under the corresponding symbols, which are presented in rows on the response sheet. This version of the coding task has been called the Symbol–Digit Substitution Test (SDST). In the present study, as in our previous studies (5, 11), we have used the SDST in order to avoid drawing unfamiliar graphic symbols, which requires a time consuming process of motor planning.

In the measurement of processing speed using a digit–symbol-coding task, at least two factors might play a considerable role. First, digit–symbol-coding tasks have a strong sensorimotor component (i.e., fine motor writing skills of the symbols or the digits). A reduction in sensorimotor speed, characterized by a longer initiation and/or execution of graphic movements, might indeed contribute substantially to low coding task performance. Previous research by Morrens et al. has demonstrated that schizophrenia patients display both sensorimotor and cognitive slowing and that these two processes are unrelated to each other (11).

In addition to a possible sensorimotor component, a second possible factor, which may influence the measurement of processing speed is het effect of (implicit) learning of the specific symbol–digit combinations. Learning is a well-known impairment in schizophrenia (9, 12–14) in addition to processing speed. Once the symbol–digit relationships are learned, it is no longer necessary to rely on visual scanning of the key on top of the administration sheet, rather working or episodic memory can be used instead for the right response. This strategy might reduce the time in finding the right response, resulting in an increased score on the test. There may be large individual differences in the speed of learning these symbol–digit relations and in their memory capacity. Similarly, Bachman et al. (15) and Joy et al. (16) proposed that a reduced cognitive processing speed in schizophrenia might be partially due to a mnemonic deficit. Other studies on this topic concluded that the contribution of memory to symbol–digit coding performance might be relatively small but relevant (16).

However, many of these previous studies have used regression-based approaches in which coding performance was correlated with additional neuropsychological tests (15). In an older version of the Wechsler Adult Intelligence Scale (WAIS-III), the Digit–Symbol-coding test was even followed by an implicit learning test to assess the recall of the symbol–digit relations (17). Bachman et al. rightly argued for a complementary experimental approach in which the symbol-coding task is manipulated to determine the role that several sub-processes might play in coding tasks. However, a disadvantage of this latter approach is that changing the task might have consequences for the relative contribution of these sub-processes.

In this experimental study, the subjects’ pen movements were recorded on a writing tablet under the test sheet in order to precisely measure the time taken to write each digit (writing time) as well as the preceding time necessary to decode a symbol into its corresponding digit (matching time). The task requires to write the digits as quickly as possible; therefore, the writing time provides an estimate of sensorimotor speed whereas matching time reflects the duration of the cognitive processes that are needed to find or recall the digit that corresponds to the stimulus symbol.

Because matching time and writing time were registered for every single digit, the decrease per symbol–digit combination offers an estimate of both the rate and the amount of learning within one (90 s) test administration. In addition, by administering the same test on three separate days, we were able to assess the amount of long-term learning of the symbol–digit relations.

Schizophrenia has been previously hypothesized as a generalized syndrome of accelerated aging (18). Since the earliest descriptions by Emil Kraepelin, schizophrenia has been referred to as “dementia praecox,” literally meaning “a cognitive decline in young age.” A number of studies have shown that processing speed as measured by the SDST is a fundamental mediator of age-related cognitive decline (19, 20). Therefore, comparing the performance of schizophrenia patients to elderly individuals could offer secondary, but valuable information as to what extent and in which domains the cognitive decline in schizophrenia resembles age-related cognitive impairment, referred to as “mild cognitive impairment.” To our knowledge, a direct comparison of performance on the SDST between schizophrenic and elderly individuals has never been conducted.

In summary, the present study was set up to investigate the relative contribution of learning and sensorimotor speed during SDST performance. Patients with schizophrenia, age-matched healthy controls, and elderly volunteers were tested in order to assess different effects of these factors in the different groups. The first hypothesis was that overall test performance would be lower in schizophrenia patients compared with age-matched healthy controls. In addition, it was expected that this study would replicate the well-known findings of reduced writing speed in schizophrenia. As visual and verbal learning and memory have been frequently found to be impaired in patients with schizophrenia (7), it was further hypothesized that the rate and amount of the learning of the symbol–digit relations would be reduced in the schizophrenia patients. The comparison of schizophrenic and elderly individuals was exploratory.

## Materials and Methods

### Study design

Our study group consisted of 30 patients with stable schizophrenia, 30 age- and sex-matched control participants, and 30 sex-matched elderly volunteers (aged 65–85 years). The SDST was administered three times on three separate assessment days. The test was conducted in accordance with the ethical principles that have their origin in the Declaration of Helsinki and that are consistent with Good Clinical Practices, applicable regulatory requirements, and in compliance with the study protocol. This study was held at the University Psychiatric Hospital Duffel, Belgium, and the study protocol was reviewed and approved by the institute’s Ethics Committee.

### Participants

The previously mentioned test subjects were recruited from the local community. Prior to the start of the study, they all provided a written informed consent and their eligibility for this study was assessed according to some inclusion and exclusion criteria. The inclusion criteria for patients were: (1) being an in- or outpatient with schizophrenia or schizoaffective disorder (DSM-IV), (2) having a known history of schizophrenia for at least 12 months, confirmed by the treating psychiatrist, and (3) receiving stable antipsychotic drug therapy (maximally 2) for at least 6 weeks prior to screening. The inclusion criteria for all participants were: (1) being a man or woman between 18 and 55 years old (schizophrenia patients and young controls) or between 65 and 85 years old (elderly volunteers) and (2) being medically stable.

The exclusion criteria applicable to all participants were (1) having a DSM-IV diagnosis of substance dependence or abuse within 3 months prior to screening evaluation (only caffeine dependence was not exclusionary), (2) use of benzodiazepines, tricyclic antidepressants, or anticholinergic medication, (3) having a positive urine screen for drug abuse or a positive alcohol breath test at screening on one of the test days, (4) having a clinically significant acute illness within 7 days prior to screening. Since the use of alcohol and drugs could potentially influence the study data, an alcohol breath test and a urine drug screen were performed before the start of each assessment day.

### Symbol–Digit Substitution Test

The task was performed on two subsequent sessions (day 1 and day 2) and a third time (day 7). The SDST was the first task to be performed on every assessment day in a larger series of cognitive tests, which will be published elsewhere. In order to avoid influences of the circadian rhythm, also the time on which the test was administered was comparable for each subject.

The coding task required to translate 9 different symbols into the digits 1–9 on five rows each consisting of 25 symbols, according to a key of symbol–digit pairs, which was presented on top of the task sheet. The same symbol–digit combinations were repeated over the three session days. In line with our previous studies (5, 11, 21), we used the reversed version of the classical DSST where the digits had to be written under the symbols, denoted by SDST. We chose for this design in order to exclude the complication of processes of motor planning by the drawing of complex graphic symbols on SDST performance. The nine different symbols were presented in blocks. The sequence in which they were presented within each block was randomized.

A quiet environment was chosen to perform this task. The participants were asked to decode the list of symbols one by one as fast as possible within a preset 90s limit, based on the key above, writing the correct digit under the corresponding symbol on a sheet of paper placed on a digitizing tablet (WACOM1218RE) with a special pressure-sensitive normal-looking ballpoint pen. Pen position was recorded at 200 Hz and with 0.2 mm spatial accuracy, and stored on a standard personal computer. The signals were subsequently filtered by means of a fast-Fourier analysis. These digitized recordings allowed the computation of separate matching- and writing times.

Identical instructions were repeated each day, before the start of the task. Feedback was not provided at the end of the session. All subjects had to undergo a practice trial on the first assessment day, consisting of filling in the last 10 symbol–digit pairs, allowing them to get familiar with the experiment.

### Statistical analysis

All data were analyzed using a general linear model (GLM) repeated measures in IBM^®^SPSS^®^ Version 22. First, we analyzed the Session effect (long-term learning) with Group (three levels) as the between-subjects variable and Session (or days, with three levels) as the within-subjects variable. A second analysis used Block (five levels; short-term learning) and Session (three levels) as the within-subject variables and Group (three levels) as the between-subject variable. We performed separate analyses for (1) the number of correct digits, (2) the mean matching time per digit, and (3) the mean writing time per digit. Wilk’s Lambda was used in the tests of the within-variable effects. A *p*-value of <0.05 was considered significant.

The number of blocks that could be analyzed depended on the lowest test score (number correct) obtained by the participants. Matching and writing time were analyzed over five blocks (i.e., number correct is 45 or higher). This score was gained in all three sessions by 25 patients with schizophrenia, 28 elderly volunteers, and 28 controls. Including more participants in the analysis would result in too many missing values in the fourth and fifth block whereas analyzing more than five blocks would result in an unrepresentative low number of participants. Per block, the median matching time and median writing time were calculated. Only correct digits were analyzed and the first digit of a row was eliminated from analysis because the transport distance to this location was more than 20 cm instead of the normal 0.8 cm. In session 3, the data of one patient were missing.

## Results

The main objective of the present study was to evaluate the role of learning processes during SDST performance. Firstly, demographics will be prescribed (see Demographics) followed by their general test scores on the SDST [see Test score (Number of correct digits)]. Matching time and writing time of test scores were separately calculated (see Long-Term Learning Matching and Writing Time and Short-Term Learning Matching and Writing Time Over Blocks Per session), and the added effects of long-term learning (section Long-Term Learning Matching and Writing Time) and short-term learning (see Short-Term Learning Matching and Writing Time Over Blocks Per session) were assessed. In a final section (see Estimating the Effect of Short-Term Learning on the SDST Score), the relative contribution of short-term learning on the overall SDST score was calculated.

### Demographics

The demographic features of the three study groups are shown in Table 1. All patients used antipsychotic medication at the time of testing. Sixteen schizophrenia patients were using more than one antipsychotic drug. The distribution of the different antipsychotic drugs and range of daily doses are summarized elsewhere (22). Seven young controls, two schizophrenia patients, and no elderly individuals were left-handed. A GLM repeated measures analysis was conducted on the performance of the SDST (number correct) for the young control group with “Session” as within-subject variable and “Handedness” as between-subject variable. “Handedness” did not have a significant influence on the overall test score. The schizophrenia group had a lower mean IQ, as measured by the Adult Reading Test/ART (Dutch version: Nederlandse Leestekst voor Volwassenen/NLV), than the control group (*t* = 3.96, *p* = 0.0002) and the elderly group (*t* = 4.71, *p* < 0.0001).

**Table 1:** **Demographics**.

		Schizophrenia	Elderly	Control
		
*N*		30	30	30
Age	Mean (SD)	36.43 (7.83)	69.33 (3.89)	36.77 (8.55)
	Range	23–53	65–79	18–52
IQ (ART)	Mean (SD)	101.3 (10.30)	111.7 (6.43)	110.1 (6.39)
	Range	66–115	100–124	98–130
Sex	male: female	2:1	2:1	2:1
Race	Asian	0	0	1
	Maghreb	1	0	0
	White	29	30	29

### Test score (number of correct digits)

**Figure 1:**
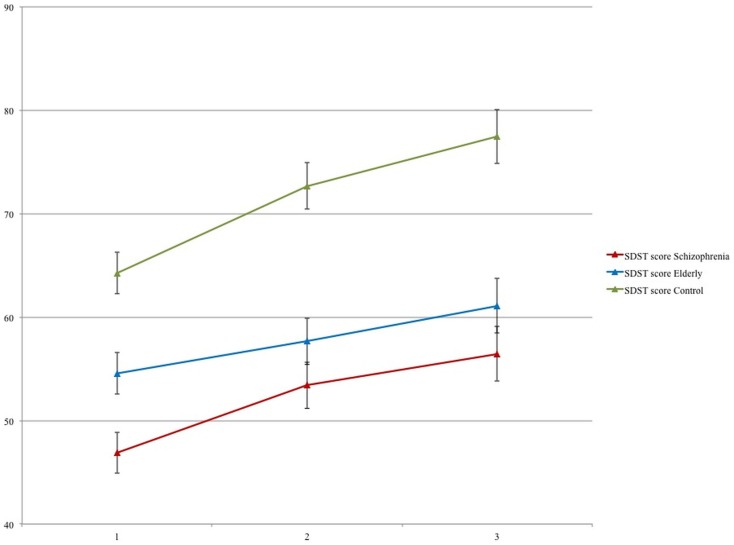
**Means and SE of the number of correct digits per session for schizophrenia patients, elderly volunteers, and young controls**.

The mean number of correct digits per session is displayed in Figure 1 for each group. This figure clearly shows an increase in task performance (long-term learning effect) over the three sessions, which was significant [*F*(2,85) = 36.21, *p* < 0.001]. This learning effect was about equal in the three groups (Session*Group interaction *p* = 0.119).

On average, the three groups differed significantly in their overall score [*F*(2,86) = 21.69, *p* < 0.001]. Both schizophrenia patients and the elderly volunteers achieved a lower test score than the controls (*p* < 0.001). Figure 1 gives the impression that the schizophrenia group performed even worse than the elderly, but the difference between these groups was not significant (*p* = 0.07) but this was only true during the first session (*p* = 0.028). After incorporating IQ as a covariate in the analysis, the group difference between schizophrenia patients and controls remained significant [*F*(1,56) = 23.61, *p* < 0.0001], but the difference between schizophrenia and the elderly on session 1 was reduced to non-significance [*F*(1,57) = 0.60, *p* = 0.443].

### Long-term learning matching and writing time

**Figure 2:**
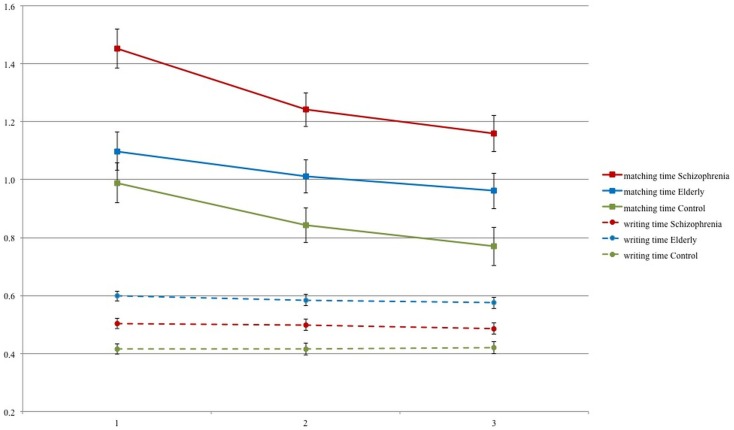
**Means and SE for matching and writing time per session for schizophrenia patients, elderly volunteers, and young controls**.

Mean matching time per digit and mean writing time per digit are presented in Figure 2 for each session and each group.

#### Mean matching time per session

Figure 2 demonstrates that the matching times mirror the SDST performance of Figure 1. A clear learning effect over sessions was found [*F*(2,85) = 32.46, *p* < 0.0001], which was equal for the three groups [Group*Session interaction: *F*(2,85) = 1.49, *p* = 0.206]. Averaged over all sessions, the matching times in each group differed significantly from each other [*F*(2,86) = 13.39, *p* < 0.001]. Planned contrasts show a significant difference between patients and controls (*p* < 0.0001), between patients and elderly (*p* = 0.002 after Bonferroni correction), but not between the elderly and the controls (*p* = 0.167). IQ (ART) as a covariate was significant [*F*(1,85) = 14.50, *p* = 0.0003]. IQ did not influence the difference between patients and controls (*p* = 0.001) but reduced the difference between elderly and schizophrenic participants to non-significance (*p* = 0.282).

#### Mean writing time per session

The writing times as displayed in Figure 2 do not show much variation over the test sessions, and the session effect was not significant [*F*(2,85) = 1.36, *p* = 0.262]. Therefore, we may conclude that there was no evident learning in the writing of the digits. Neither was the Group*Session interaction significant [*F*(4,170) = 0.45, *p* = 0.771]. On the other hand, the differences between the groups were relatively large and significant [*F*(2,86) = 26.37, *p* < 0.0001]. The elderly wrote significantly slower than the patients (*p* = 0.0003) and the patients wrote significantly slower than the controls (*p* = 0.001).

### Short-term learning matching and writing time over blocks per session

**Figure 3:**
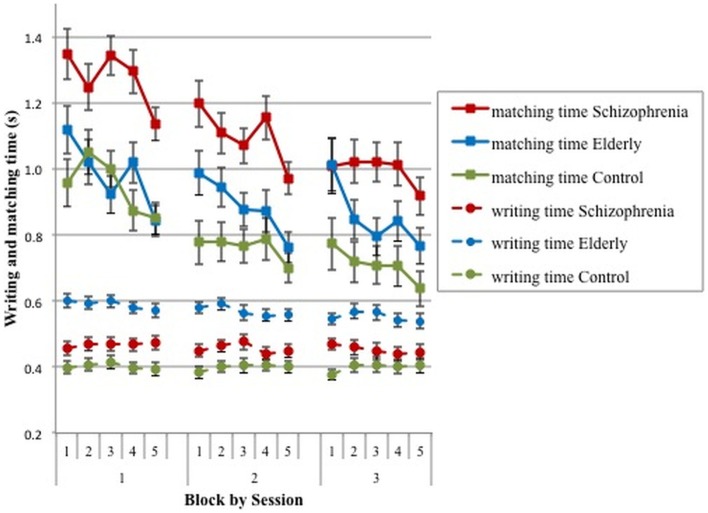
**Means and SE for writing and matching time per block and per session for schizophrenia patients, elderly volunteers, and young controls**.

Within-session learning effects are shown in Figure 3, which displays mean matching and writing times per digit for each of the five blocks in the three sessions.

#### Mean matching time per block

Figure 3 illustrates a decrease in matching time over the blocks and over sessions. A GLM repeated measures analysis confirmed that matching time decreased significantly over blocks [short-term learning; *F*(4,75) = 21.66, *p* < 0.0001] and over sessions [long-term learning; *F*(4,75) = 21.66, *p* < 0.0001]. The decrease over blocks was about equal in the three sessions [*F*(8,71) = 1.74, *p* = 0.105] and seemed to be similar in the three groups [*F*(8,150) = 1.709, *p* = 0.101], but the highest order interaction (session*block*group) was significant [*F*(16,142) = 1.79, *p* = 0.038]. Therefore, separate analyses were run per session. In these analyses, only the linear block effect was tested (i.e., a linear decrement of matching time over blocks; see the dashed lines in Figure 3). In session 1, this linear block effect was significant [*F*(1,78) = 31.04, *p* < 0.0001], denoting a significant short-term learning effect (decrement over blocks), but this learning effect was similar for the three groups [block*group Linear: *F*(2,78) = 0.03, *p* = 0.597]. In the second and third sessions, more participants reached the minimum criterion of 45 correct digits, but the analyses of the Linear trends yielded similar results [session 2: block: *F*(1,83) = 29.08, *p* < 0.0001; block*group Linear: *F*(2,83) = 2.75, *p* = 0.070; session 3: block: *F*(1,83) = 25.20, *p* < 0.0001; block*group Linear: *F*(2,83) = 1.95, *p* = 0.148]. Therefore, these results suggest that the rate and amount of short-term learning (repetition over blocks) was similar in the three sessions and about equal among the three groups.

#### Mean writing time per block

Writing time in Figure 3 shows that there is not much variation over blocks and sessions. The only noteworthy result is the relatively long writing time of the elderly participants.

An analysis of writing time with session and block as the within-subject variables and group as the between-subject variable showed that the effect of session was not significant but the block effect was [*F*(4,75) = 5.60, *p* = 0.0003]. None of the interactions were significant either. In the first session, the linear block effect was not significant (*p* = 0.216), but the linear block*group interaction was significant [*F*(2,78) = 3.55, *p* = 0.033]. This is probably due to a slight decrement of writing time by the elderly but not by the other two groups. In the second and third session, the linear block effect and the linear block*group interactions were not significant, indicating that writing time remained stable in these sessions.

### Estimating the effect of short-term learning on the SDST score

To estimate the contribution of the linear decrease in matching time to the SDST score, we estimated the score that would have been obtained if the matching time (mt) and writing time (wt) of the first block (i.e., mt1 and wt1) had been maintained over all blocks in the 90 s of the test [i.e., estimated score = 90/(mt1 + wt1)]. We did the same for block five. The result of this estimation for session 1, i.e., for the standard test administration, was that the score of all participants had improved, more specifically from 50 to 56 for patients with schizophrenia, from 52 to 64 for the elderly and from 66 to 72 for the controls. These are increases of 12, 23, and 9%, respectively. It should, however, be stipulated that not all participants showed a decrease in matching time over blocks. Slopes ranged considerably, with the largest range found in the group of schizophrenia patients (from +78 ms/block to −301 ms/block; mean = −37 ms/block) compared to the ranges of the young controls (from + 50 ms/block to −284 ms/block; mean = −39 ms/block) and the elderly volunteers (from + 38 ms/block to −236 ms/block; mean = −55 ms/block).

## Discussion

### Summary of results

The main purpose of this study was to assess to what extent differences in symbol–digit learning influence the performance on the SDST. The present findings demonstrate that the repetition of symbol–digit pairs during one test administration (short-term learning), and the repetition of the same test over several days (long-term learning), resulted in significant decreases of matching time. Interestingly, these learning effects on matching time were about equal for patients, age-matched controls, and elderly participants, while the overall test score differed among the groups. In contrast, writing time, reflecting sensorimotor speed, remained about equal over symbol–digit repetitions. Patients had the lowest overall score and the longest matching time; however, the difference between patients with schizophrenia and elderly was no longer significant after controlling for the lower IQ of the patients. Sensorimotor speed had a smaller impact on the overall test performance, but there were significant differences between the three groups with the elderly clearly being the slowest writers.

### Rationale for the chosen methodological approach

In an experimental approach of the coding task, like the one adopted by Bachman et al. (15), single symbol–digit pairs are presented trial by trial and on each trial the participant has to quickly decide whether the presented combination is identical to one of the digit–symbol pairs in the reference code that is simultaneously presented on the PC screen. In the more common paper-and-pencil version of the task, the participant can work at his own pace and might (learn to) combine the activities of both writing a digit and searching for the next digit that matches the next symbol in parallel. We opted to incorporate an experimental approach into the continuous paper-and-pencil version, because recording of the pen movements enables the separate measurement of reaction time (now denoted by “matching” time) and response execution time (“writing” time).

In addition, to allow an unbiased estimate of learning we adapted the presentation of the symbols that had to be coded. In standard symbol-coding tests, not all nine symbols are already shown in the first block but they are introduced gradually to promote the learning of the symbol–digit relations. For our SDST version, however, we preferred to present all nine symbols with the same frequency right from the start. As a result, a repetition of the same symbol–digit pair was separated by an average of eight other pairs. Yet, considerable learning did occur as evidenced by the linear decrease in matching time over nine-symbol blocks.

### Influence of learning processes on the SDST test score

Comparing the size of the learning effects with the SDST scores showed that the influence of learning processes on the SDST score in schizophrenia, the elderly and younger controls varies greatly from person to person. The average learning effects found in the present study of about 12% in the schizophrenia group and 23% in the elderly can be classified as rather substantial. This is in line with the conclusions drawn by Bachman et al. (15) and Joy (16). It deviates from Salthouse’s (23) interpretation in which memory factors are assigned only “a very small role in contributing to the age decline in digit–symbol performance.”

### Identification of learning processes

Repetition of the same task results in learning. Therefore, the decrease in matching time over blocks within one session as well as over more sessions must be the result of a learning process. But what exactly is learned during the repetitions of symbol–digit pairs is less clear. Two critical processes are known to be involved in the search for the matching digit: visual scanning (24) and relational memory (16). First, visual scanning, refers to the early detection and identification of visual stimuli, either alone or in the presence of competing stimuli. The role of visual scanning is emphasized when participants consult the code key frequently during test administration. Possibly, visual scanning might improve by learning the position of the symbols in the key. Second, relational memory, refers in this context to the memory for associations in the SDST (10, 12). Learning the relations between symbols and digits will reduce the necessity for searching, which automatically results in a decreased matching time. A third process that might be involved in the reduction of matching time over repetitions is a change in the strategy to perform the task. An impairment in response selection (25, 26), i.e., the process of mapping stimuli to specific responses and decision making could possibly cause a considerable amount of the lower performance observed in schizophrenia. Most participants will start with performing matching and writing strictly after each other, while some participants might learn to do part of the writing and scanning in parallel. In that case, the search for the next digit has already started during the more or less automatic writing of the current number. Overall, various learning processes might contribute to a decreased matching time, but their relative contribution could not be deduced from the present study. To find more detailed answers, experimental changes of the task, like the manipulations tested by Bachman et al., are needed. For now, we can only conclude that these learning processes occurred unintentionally, and therefore, should be denoted by the term “implicit learning.” An important outcome of this study is that the rate of this implicit learning was not significantly different among the three groups.

### Sensorimotor speed

The second aim of our investigation was to evaluate the effect of differences in writing speed on the SDST score. Group differences in writing speed were highly significant but smaller than the group differences in matching time. Schizophrenia patients wrote significantly slower than same-aged controls and the elderly had the lowest writing speed. The effects of reduced writing speed in schizophrenia and the elderly on the total test score were smaller than the estimated effects of learning (for schizophrenia, learning + 12%, writing speed + 4%; for the elderly, learning + 23%, writing speed + 13%). This leads us to the conclusion that the usual determination of the symbol-coding test score results in underestimation of the speed of information processing, particularly for the elderly.

### Schizophrenia and the elderly compared

Healthy elderly persons were included in this study in order to compare the reduction in the speed of information processing of the schizophrenia patients with normal, age-related cognitive decline. By correcting for sensorimotor speed and only taking the matching time as an index of processing speed, patients performed worse compared to the elderly volunteers (aged 65–85 years). However, when an estimate of premorbid intelligence (the Adult Reading Test) was taken into account, the differences in matching time were no longer statistically significant, while the difference between patients and same-aged controls still remained significant. Although the similarity between the elderly and the patients with schizophrenia on matching time is striking, we cannot deduce from these data whether schizophrenia should be seen as “dementia praecox.” In addition, it should be acknowledged that the elderly had a small but significantly lower sensorimotor speed. Only sensorimotor speed differentiated all three groups.

### Study limitations

Due to patient selection, a bias might exist since only patients who were able to complete the test batteries were included in this study. The neurocognitive abilities of the selected patients may therefore be higher than the group of schizophrenia at large. Thus, the results of this study may not be generalized to the whole population of patients with schizophrenia. However, the mean SANS score for schizophrenia patients of 25.7 (SD 17.39) that was measured on screening visit is comparable with the mean SANS score of 23.0 (SD 14.6) found in a large heterogeneous sample of schizophrenia patients (27). Additionally, only 4.2% of our study sample was excluded after screening visit, suggesting that the internal validity of our study is high.

### Implications for future research and clinical practice

There is a wide variation in the administration of symbol–digit coding tasks ranging from a classical pen-and-paper writing task to a purely computerized test, which simply requires pressing a button when the correct symbol–digit combination appears. These different methods may ask for different cognitive sub-processes in the total test score. As an example of this, the present study clearly showed that the time taken by the motor part of the test must be taken into account in interpreting symbol–digit coding test scores as measures of the speed of information processing. The large variation between individual participants in the rate of short-term learning could argue for the need of additional memory test information to assess to what extent the (possibly) low SDST score has been the result of a learning failure. Some healthy volunteers mentioned spontaneously at the end of a session that they had remembered the symbol–digit combinations but we did not give a questionnaire to draw further conclusions toward awareness differences among the groups. Therefore, we suggest that the addition of a self-rater or observer-rater questionnaire might be valuable to address the possibility of different explicit and implicit learning strategies.

Although schizophrenia is often characterized by a reduced speed of information processing, the present study showed a similarity with the control group and the elderly as far as the rate and amount of both short-term and long-term implicit learning was involved. This was found despite the general finding of impaired working memory and a lower rate of explicit verbal and visual learning in schizophrenia. Because we speculate that improving processing speed may be predictive for the functional outcome, we recommend that more attention should be paid to implicit learning in future schizophrenia research and in the design of specific rehabilitation programs.

## Conclusion

We can conclude that the two factors that were studied both had an effect on the estimation of processing speed with the Symbol–Digit Substitution Test. The average effect of learning the symbol–digit relation on the SDST score was substantial and the large individual differences in the amount of learning deserves more attention. The effects of sensorimotor speed on the total test score were shown to be smaller than the learning effects, but cannot be neglected because they lead to an underestimation of the speed of information processing, particularly for the elderly.

The finding of equal unintentional learning effects in patients, their age-matched controls and elderly participants lead us to the conclusion that implicit learning might be preserved in schizophrenia. This finding has important consequences for the design of specific rehabilitation programs for schizophrenia patients.

## Author Contributions

All authors met ICMJE criteria and all those who fulfilled those criteria were listed as authors. All authors had access to the study data and made the final decision about where to present these data.

## Conflict of Interest Statement

This study was funded by Janssen Research and Development, a division of Janssen Pharmaceutica N.V. Mr. Janssens and Mr. Timmers are employees of Janssen Research and Development, a division of Janssen Pharmaceutica N.V., Beerse, Belgium, and own stock/stock options in the company.

## References

[1] Weinberger DR Gallhofer B Cognitive function in schizophrenia. Int Clin Psychopharmacol (1997) 4:29–36 10.1097/00004850-199709004-00006 9352344

[2] Nuechterlein KH Ventura J Subotnik KL Bartzokis G. The early longitudinal course of cognitive deficits in schizophrenia. J Clin Psychiatry (2014) 75:25–9. 10.4088/JCP.13065.su1.06 24919168 PMC4081490

[3] Nuechterlein KH Barch DM Gold JM Goldberg TE Green MF Heaton RK. Identification of separable cognitive factors in schizophrenia. Schizophr Res (2004) 72:29–39. 10.1016/j.schres.2004.09.007 15531405

[4] Brébion G Amador X Smith MJ Gorman JM Memory impairment and schizophrenia: the role of processing speed. Schizophr Res (1998) 30:31–9 10.1016/S0920-9964(97)00123-0 9542786

[5] Morrens M Hulstijn W Matton C Madani Y van Bouwel L Peuskens J Delineating psychomotor slowing from reduced processing speed in schizophrenia. Cogn Neuropsychiatry (2008) 13:457–71. 10.1080/13546800802439312 19048439

[6] Pashler H. Dual-task interference in simple tasks: data and theory. Psychol Bull (1994) 116:220–44. 10.1037/0033-2909.116.2.220 7972591

[7] Nuechterlein KH Green MF Kern RS Baade LE Barch DM Cohen JD The MATRICS consensus cognitive battery, part 1: test selection, reliability, and validity. Am J Psychiatry (2008) 165:203–13. 10.1176/appi.ajp.2007.07010042 18172019

[8] Dickinson D Ramsey ME Gold JM. Overlooking the obvious: a meta-analytic comparison of digit symbol coding tasks and other cognitive measures in schizophrenia. Arch Gen Psychiatry (2007) 64:532–42. 10.1001/archpsyc.64.5.532 17485605

[9] Knowles EEM David AS Reichenberg A. Processing speed deficits in schizophrenia: reexamining the evidence. Am J Psychiatry (2010) 167:828–35. 10.1176/appi.ajp.2010.09070937 20439390

[10] Green MF Nuechterlein KH Gold JM Barch DM Cohen J Essock S Approaching a consensus cognitive battery for clinical trials in schizophrenia: the NIMH-MATRICS conference to select cognitive domains and test criteria. Biol Psychiatry (2004) 56:301–7. 10.1016/j.biopsych.2004.06.023 15336511

[11] Morrens M Hulstijn W Van Hecke J Peuskens J Sabbe BGC. Sensorimotor and cognitive slowing in schizophrenia as measured by the symbol digit substitution test. J Psychiatr Res (2006) 40:200–6. 10.1016/j.jpsychires.2005.04.014 16039670

[12] Horan WP Green MF Knowlton BJ Wynn JK Mintz J Nuechterlein KH Impaired implicit learning in schizophrenia. Neuropsychology (2008) 22:606–17 10.1037/a0012602 18763880 PMC2548320

[13] McDowd J Tang T-C Tsai P-C Wang S-Y Su C-Y. The association between verbal memory, processing speed, negative symptoms and functional capacity in schizophrenia. Psychiatry Res (2011) 187:329–34. 10.1016/j.psychres.2011.01.017 21320727

[14] Morrens M Hulstijn W Sabbe B Psychomotor slowing in schizophrenia. Schizophr Bull (2007) 33:1038–53 10.1093/schbul/sbl051 17093141 PMC2632327

[15] Bachman P Reichenberg A Rice P Woolsey M Chaves O Martinez D Deconstructing processing speed deficits in schizophrenia: application of a parametric digit symbol coding test. Schizophr Res (2010) 118:6–11. 10.1016/j.schres.2010.02.1029 20194004 PMC2900389

[16] Joy S Fein D Kaplan E. Decoding digit symbol: speed, memory, and visual scanning. Assessment (2003) 10:56–65. 10.1177/0095399702250335 12675384

[17] Lezak MD Howieson DB Loring DW Neuropsychological Assessment. Fourth Edition. New York: Oxford University Press (2004).

[18] Kirkpatrick B Messias E Harvey PD Fernandez-Egea E Bowie CR. Is schizophrenia a syndrome of accelerated aging? Schizophr Bull (2008) 34:1024–32. 10.1093/schbul/sbm140 18156637 PMC2632500

[19] Crawford JR Bryan J Luszcz MA The executive decline hypothesis of cognitive aging: do executive deficits qualify as differential deficits and do they mediate age-related memory decline? Aging Neuropsychol Cogn (2000) 7:9–31 10.1076/anec.7.1.9.806

[20] Baudouin A Clarys D Vanneste S Isingrini M. Executive functioning and processing speed in age-related differences in memory: contribution of a coding task. Brain Cogn (2009) 71:240–5. 10.1016/j.bandc.2009.08.007 19796862

[21] Morrens M Hulstijn W Sabbe B. The effects of atypical and conventional antipsychotics on reduced processing speed and psychomotor slowing in schizophrenia: a cross-sectional exploratory study. Clin Ther (2008) 30:684–92. 10.1016/j.clinthera.2008.04.012 18498917

[22] De Picker L Cornelis C Hulstijn W Dumont G Fransen E Morrens M Preserved sensorimotor learning in stable schizophrenia patients but not in elderly participants compared to young controls in two variations of the Rotary Pursuit. Front Psychiatry (2014) 5:165 10.3389/fpsyt.2014.00165 25505425 PMC4241745

[23] Salthouse TA. The role of memory in the age decline in digit-symbol substitution performance. J Gerontol (1978) 33:232–8. 10.1093/geronj/33.2.232 637915

[24] Mahurin RK Velligan DI Miller AL. Executive-frontal lobe cognitive dysfunction in schizophrenia: a symptom subtype analysis. Psychiatry Res (1998) 79:139–49. 10.1016/S0165-1781(98)00031-6 9705052

[25] Woodward ND Duffy B Karbasforoushan H. Prefrontal cortex activity during response selection predicts processing speed impairment in schizophrenia. J Int Neuropsychol Soc (2013) 19:782–91. 10.1017/S1355617713000532 23816240 PMC3910268

[26] Krieger S Lis S Janik H Cetin T Gallhofer B Meyer-Lindenberg A. Executive function and cognitive subprocesses in first-episode, drug-naive schizophrenia: an analysis of N-back performance. Am J Psychiatry (2005) 162:1206–8. 10.1176/appi.ajp.162.6.1206 15930072

[27] van Erp TG Preda A Nguyen D Faziola L Turner J Bustillo J Converting positive and negative symptom scores between PANSS and SAPS/SANS. Schizophr Res (2014) 152:289–94. 10.1016/j.schres.2013.11.013 24332632 PMC3966195

